# Readability analysis of ChatGPT's responses on lung cancer

**DOI:** 10.1038/s41598-024-67293-2

**Published:** 2024-07-26

**Authors:** Adem Gencer

**Affiliations:** Department of Thoracic Surgery, Faculty of Medicine, Afyonkarahisar Health Sciences University, Zafer Sağlık Külliyesi, Dörtyol Mah. 2078 Sok. No:3 A Blok Afyonkarahisar, Afyonkarahisar, Turkey

**Keywords:** Lung cancer, Artificial intelligence, Large language models, ChatGPT, Readability, Comprehensibility, Cancer, Computational biology and bioinformatics, Medical research

## Abstract

For common diseases such as lung cancer, patients often use the internet to obtain medical information. As a result of advances in artificial intelligence and large language models such as ChatGPT, patients and health professionals use these tools to obtain medical information. The aim of this study was to evaluate the readability of ChatGPT-generated responses with different readability scales in the context of lung cancer. The most common questions in the lung cancer section of Medscape^®^ were reviewed, and questions on the definition, etiology, risk factors, diagnosis, treatment, and prognosis of lung cancer (both NSCLC and SCLC) were selected. A set of 80 questions were asked 10 times to ChatGPT via the OpenAI API. ChatGPT's responses were tested using various readability formulas. The mean Flesch Reading Ease, Flesch-Kincaid Grade Level, Gunning FOG Scale, SMOG Index, Automated Readability Index, Coleman-Liau Index, Linsear Write Formula, Dale-Chall Readability Score, and Spache Readability Formula scores are at a moderate level (mean and standard deviation: 40.52 ± 9.81, 12.56 ± 1.66, 13.63 ± 1.54, 14.61 ± 1.45, 15.04 ± 1.97, 14.24 ± 1.90, 11.96 ± 2.55, 10.03 ± 0.63 and 5.93 ± 0.50, respectively). The readability levels of the answers generated by ChatGPT are "collage" and above and are difficult to read. Perhaps in the near future, the ChatGPT can be programmed to produce responses that are appropriate for people of different educational and age groups.

## Introduction

Lung cancer is one of the most common malignant tumors, with high morbidity and mortality^1^. The five-year survival rate for individuals diagnosed with lung cancer is typically reported to be between 10 and 20%^2,3^. As in many diseases, the internet is a popular platform to access information on lung cancer today^4^. The tendency of patients to search for answers to health-related questions on the internet is increasing day by day. While search engines are often used for this purpose, artificial intelligence tools such as GPT (Generative Pre-trained Transformer) are increasingly being used for this purpose as a result of developments in technology ^4^.

Large language models (LLMs) are algorithms that can detect and analyze natural language and generate unique responses and are new developments in artificial intelligence and neural networks^5^. OpenAI (San Francisco, CA) developed ChatGPT, one of the most well-known LLMs today^6^. Since its initial debut in November 2022, it has, on average, added 25 million users by February 2023. ChatGPT's generative powers set it apart from other AI solutions^7^. ChatGPT is a promising technology that has the potential to revolutionize the healthcare industry, including pharmacy, by offering practitioners, students, and researchers’ most up-to-date medical information and support in a conversational, interactive manner^8^.

ChatGPT's ability to provide accurate and fast answers to complex health questions has attracted the interest of many researchers, and many studies are planned to examine the potential of ChatGPT on medical-related topics. Previous research has shown that ChatGPT can be successful in medical exams^9,10^. Some researchers have mentioned the advantages of ChatGPT in medical article writing^6,11,12^. ChatGPT also provides diagnosis and treatment recommendations to patients and healthcare professionals regarding medical issues^13–15^. Therefore, ChatGPT is being used, researched, and tested by more and more people in this field.

Undoubtedly, the accuracy and reliability of ChatGPT's answers to health-related questions are extremely important. Several studies have been documented in the academic literature pertaining to this particular topic^14,16,17^. Nevertheless, the readability and comprehensibility of the responses generated by ChatGPT are equally significant factors to consider. The aim of this study was to evaluate the readability of ChatGPT-generated responses with different readability scales in the context of lung cancer.

## Material and methods

This article does not contain any studies with human or animal subjects, and ethical approval is not applicable for this article.

For this study, the most common questions in the lung cancer section of Medscape^®^ (WebMD LCC, US) were reviewed, and 80 questions on the definition, etiology, risk factors, diagnosis, treatment, and prognosis of lung cancer (both NSCLC and SCLC) were selected. Medscape^®^ is a leading online global destination for physicians and healthcare professionals worldwide, offering the latest medical news and expert perspectives; essential point-of-care drug and disease information; and relevant professional education and CME.

A python code specially prepared for this study was used to transmit the questions to ChatGPT and receive the answers. The answers were obtained through English version of ChatGPT-API, supported by the "gpt-3.5-turbo" model provided by OpenAI^®^. Each question was asked to ChatGPT 10 times in total, and 10 answers were obtained. The Python code was run in a single run on October 1, 2023. A total of 800 answers obtained for 80 questions were exported to a file (Supplementary Material 1) and analyzed for readability.

### Readability formulas

#### Flesch Reading Ease (FRE) formula

Rudolph Flesch developed the Flesch Reading Ease (FRE) formula in 1948. The FRS ranges from 1 to 100, where 100 is the highest level of readability. A score of 60 is considered standard for publications targeting a general audience, and a score of 70 or more is considered easy for the average adult to read^18^.

#### flesch-kincaid grade level (FKGL)

The Flesch Reading Grade Level formula was built upon in FRE by Kincaid et al. in 1975 for the US Navy to give a grade level to written material. It is commonly referred to as the Flesch–Kincaid Grade Level (FKGL). Both FRE and FKGL calculate the readability based on two variables: average sentence length (based on the number of words) and average word length (based on the number of syllables)^19^.

#### Fog scale (gunning FOG formula)

The Gunning Fog Index is a readability formula that estimates the years of formal education required to understand a piece of text on the first reading^20^. It is based on the average number of words per sentence and the percentage of complex words in the text. The formula calculates the grade level at which the text is written, with a higher grade level indicating more complex and difficult-to-understand text^21^.

#### SMOG index

The Simplified Measure of Gobbledygook (SMOG) index is a readability formula used to assess the readability of a piece of text. It estimates the years of education required to understand the text on the first reading^22^. The SMOG index takes into account the number of polysyllabic words in a sample of text and uses a formula to calculate the grade level at which the text is written^21^.

#### Automated readability index (ARI)

The Automated Readability Index (ARI) is a readability formula used to assess the readability of a piece of text. It estimates the years of education required to understand the text on the first reading. The Automated Readability Index (ARI) considers the mean number of characters per word and the mean number of words per sentence within a given text sample. By employing a specific formula, the ARI determines the grade level at which the text is composed^23^.

#### Coleman-Liau index

The Coleman-Liau Index is a readability formula used to assess the readability of a piece of text. It estimates the years of education required to understand the text on the first reading. The Coleman-Liau Index is a metric that considers the mean number of characters per word and the mean number of sentences per 100 words within a given text sample. By employing a specific formula, this index determines the grade level at which the text is composed^24^.

#### Linsear write formula

The Linsear Write Formula is a readability formula used to assess the readability of a piece of text. The metric provides an estimation of the number of years of formal education necessary to comprehend the content upon initial perusal. The Linsear Write Formula considers the presence of both simple and complex words within a given text sample, employing a specific formula to determine the grade level at which the text is written^25^.

#### Dale-Chall readability score

The Dale-Chall Readability Score is a widely used formula for assessing the readability of a text. The text's grade level is determined by analyzing the frequency of complex vocabulary employed within it. This method has been utilized in numerous research endeavors to assess the comprehensibility of diverse forms of literature, encompassing materials designed for patient education, survey inquiries, and internet health-related content^26^.

#### Spache readability formula

The Spache Readability Formula is a widely employed tool for evaluating the readability of written material, with a specific focus on children's literature. The text's grade level can be determined by estimating the frequency of familiar words it contains. In honor of his wife Alice Spache, G. Harry McLaughlin created the formula, which is also known as the Spache formula ^27^.

### Statistical analysis

We used a custom code written in Python (v3.9.18) to get the responses from ChatGPT. ChatGPT communication was set up with the English version of ChatGPT-API (premium version) based on the "gpt-3.5-turbo" model provided by OpenAI^®^. The "textstat 0.7.3" python library was used to calculate readability formulas. Data analysis was performed on Python (v3.9.18) using Pandas (v1.4.4) and Numpy (v1.24.3) libraries. The results obtained from the study were presented using descriptive statistical methods (mean, standard deviation, minimum, and maximum).

## Results

The 80 questions (with 10 iterations) on diagnosis, treatment, prognosis, and risk factors of lung cancer (both SCLC and NSCLC) were asked to ChatGPT with a Python script specific to this study. It took approximately 4 h and 7 min to obtain a total of 800 responses. The mean response time for each question was 18.52 ± 5.53 s. The fastest response was 4.26 s, while the slowest response was 97.80 s.

The shortest response given by ChatGPT to questions related to lung cancer was "How frequently is tobacco smoking the cause of non-small cell lung cancer?”. The response to this question contains 4 sentences, 33 words, and 328 characters. The longest response was to the question "How is lung cancer diagnosed?" and was 23 sentences, 250 words, and 2579 characters. The mean response length is 12.95 ± 3.76 sentences, 144.25 ± 35.73 words, and 1428.76 ± 380.58 characters.

Considering the readability of all the responses given by ChatGPT, it is seen that the mean Flesch Reading Ease, Flesch-Kincaid Grade Level, Gunning FOG Scale, SMOG Index, Automated Readability Index, Coleman-Liau Index, Linsear Write Formula, Dale-Chall Readability Score, and Spache Readability Formula scores are at a high level (mean and standard deviation: 40.52 ± 9.81, 12.56 ± 1.66, 13.63 ± 1.54, 14.61 ± 1.45, 15.04 ± 1.97, 14.24 ± 1.90, 11.96 ± 2.55, 10.03 ± 0.63 and 5.93 ± 0.50, respectively). Descriptive statistics on the readability levels of all responses can be seen in Table 1. Among the reponses given by ChatGPT to the questions, the sample responses with the highest and lowest FRE scores are given in Table 2.

**Table 1:** Readability level of ChatGPT responses.

Formula	Mean ± Standard Deviation	Minimum—Maximum
Flesch reading ease	40.52 ± 9.81	6.95—69.52
Flesch-kincaid grade level	12.56 ± 1.66	7.1—18.7
Gunning FOG scale	13.63 ± 1.54	9.22—20.01
SMOG index	14.61 ± 1.45	10.1—19.5
Automated readability index	15.04 ± 1.97	8.9—22.2
Coleman-liau index	14.24 ± 1.90	8.18—20.83
Linsear write formula	11.96 ± 2.55	4.8—22.3
Dale-chall readability score	10.03 ± 0.63	7.99—13.0
Spache readability formula	5.93 ± 0.50	4.54—8.27

**Table 2:** Sample of the lowest and highest scores based on the FRE.

The response with the lowest score based on the FRE criterion FRE Score: 6.95 Question: How are the potential complications of small cell lung cancer managed?	ChatGPT Response: The management of potential complications associated with small cell lung cancer (SCLC) often involves a multidisciplinary approach that includes medical oncologists, radiation oncologists, thoracic surgeons, pulmonologists, and other specialists. The specific complications of SCLC and their management can vary, but here are some commonly encountered complications and how they may be managed: 1. Paraneoplastic syndromes: SCLC frequently produces substances that can cause paraneoplastic syndromes such as SIADH (syndrome of inappropriate antidiuretic hormone secretion) or Lambert-Eaton myasthenic syndrome. These syndromes are often treated by addressing the underlying cancer with chemotherapy, radiation therapy, or surgery, along with supportive measures specific to each syndrome 2. Pulmonary complications: SCLC can lead to various pulmonary complications, such as pneumonia, pleural effusion, or pneumothorax. Treatment depends on the specific complication but may involve antibiotics, thoracentesis (draining fluid from the pleural cavity), chest tube placement, or other interventions as necessary 3. Metastasis-related complications: SCLC frequently spreads to other organs, leading to complications depending on the involved site. The management involves a combination of systemic therapy (chemotherapy, targeted therapy, immunotherapy), radiation therapy, and occasionally surgery for localized metastases 4. Superior vena cava syndrome (SVCS): SVCS may occur due to compression or invasion of the superior vena cava by the tumor. The management of SVCS often involves urgent interventions to relieve the obstruction, such as radiation therapy, chemotherapy, stent placement, or surgery 5. Neurological complications: SCLC can metastasize to the brain, causing neurological complications such as seizures, focal deficits, or cognitive impairments. Treatment may include whole-brain radiation therapy, surgery for localized lesions, or targeted therapies for certain genetic mutations It is crucial for individuals with small cell lung cancer to receive personalized care and have regular follow-up appointments to monitor for potential complications throughout their treatment course. Treatment plans are tailored to the individual's specific situation and may involve a combination of different therapeutic modalities
The response with the highest score based on the FRE criterion FRE Score: 69.52 Question: How is non-small cell lung cancer staged?	ChatGPT Response: Non-small cell lung cancer (NSCLC) is typically staged using the TNM system, which stands for Tumor, Nodes, and Metastasis. This system is based on the extent of the tumor, whether it has spread to nearby lymph nodes, and whether it has metastasized (spread) to distant organs or tissues. The TNM system consists of several stages: 1. Tumor Stage (T): This evaluates the size and extent of the primary tumor. It can range from T1 (small tumor limited to the lungs) to T4 (large tumor involving nearby structures like the chest wall or major blood vessels) 2. Nodal Stage (N): This assesses whether the cancer has spread to nearby lymph nodes. It can range from N0 (no lymph node involvement) to N3 (cancer present in multiple lymph nodes) 3. Metastasis Stage (M): This indicates whether the cancer has spread to distant sites. M0 means no metastasis, while M1 indicates the presence of metastasis Combining these stages gives an overall stage, referred to as the TNM stage, which ranges from stage I to stage IV: - Stage I: The cancer is small and localized to the lungs, with no lymph node or distant spread - Stage II: The tumor may be larger, involve nearby structures, or have spread to nearby lymph nodes - Stage III: The tumor has spread to more lymph nodes or nearby structures - Stage IV: The cancer has metastasized to other organs or distant lymph nodes Within each stage, often subcategories like "a" and "b" are assigned, indicating further details about tumor size, lymph node involvement, or metastasis presence The staging of NSCLC is crucial for determining treatment options and predicting prognosis, helping doctors develop an appropriate treatment plan for patients

## Discussion

Today, many people, whether they are patients or not, receive information from alternative sources other than face-to-face meetings with physicians and health professionals. With the development of technology and especially the widespread use of the internet, studies have shown that a significant proportion of patients use the internet for health-related purposes, including seeking information about their conditions, treatment options, and medications^28–30^. In addition, exciting developments in artificial intelligence have enabled patients and even health professionals to add a new one to the sources of health information^31,32^.

ChatGPT is one of the most exciting technologies in today's technology world. The use and potential of this technology, which can produce answers by understanding the commands (questions) given, in the field of health is being investigated more and more every day. Large language models belonging to the natural language processing sub-branch of artificial intelligence can analyze and make sense of questions asked in natural spoken language and produce original answers very quickly. In our study, the API script using the "chatgpt-3.5-turbo" model answered the questions relatively quickly (mean response time was 18.52 ± 5.53 s). It is possible that improvements in processor, storage, and internet connection speeds could reduce this time even further.

The most important feature of artificial intelligence and natural language models is that they produce original responses using natural language. Although it is the nature of the system to be authentic, the authenticity of the responses produced by ChatGPT has been investigated in many studies in the literature^33,34^.

The fast and unique responsiveness of ChatGPT will be useless if it cannot produce accurate and reliable answers. Especially in the field of health, ChatGPT is expected to be much more reliable. Providing false, incomplete, or misleading information through ChatGPT and similar artificial intelligence applications will significantly affect the health of patients. For example, if patients are not given accurate information about lung cancer, there may be a delay in diagnosis, and the patient may miss the chance of an early diagnosis. Moreover, inaccurate information in treatment protocols may affect the decisions of healthcare professionals who are supported by artificial intelligence applications such as ChatGPT while creating diagnosis and treatment strategies. For this reason, many studies have investigated how accurately ChatGPT can produce answers to health-related questions^35–38^. Many studies have been published on how successful ChatGPT can be in exams for medical students, physicians, and health professionals^10,39–41^. Although it has been suggested that chatGPT can be successful in medical exams, there are some studies in the literature that argue the opposite^42^.

ChatGPT's ability to produce fast and original answers that are also accurate and reliable is, of course, a great achievement. ChatGPT and many other artificial intelligence tools are used by many people of very different ages and education levels. The fact that these tools do not require any additional cost other than an internet connection and provide more natural responses allows them to be used by many people. For example, a smoker may want to investigate etiological issues related to lung cancer. Or a person whose radiology report shows a nodule or mass may want to find out the stage of his or her cancer before consulting his or her physician. In addition, of course, medical students and other health sciences students, healthcare professionals, physicians, and those who provide professional healthcare services also benefit from this service offered by artificial intelligence. As a result, there is a group with a very different level of education and age. Therefore, in a disease with a high mortality rate, such as lung cancer, it is extremely important that ChatGPT not only provides correct answers but also provides readable and understandable answers. To address this aspect of ChatGPT, we investigated several readability scores accepted in the literature.

The most commonly used formulas for readability testing are Flesch Reading Ease (FRE) and Flesch-Kincaid Reading Grade Level (FKGL). According to the FRE score, the most comprehensible response produced by the ChatGPT was at the "standard" level, while the most incomprehensible response was at the "very confusing" level (69.52 and 6.95, respectively). In FKGL, the lowest score was 7.1 and the highest score was 18.7 ("professional" level and "college graduate" level, respectively). A study of urology patients found that the readability level of ChatGPT responses was similarly low according to the FRE and FKGL formulas (median 18, 15.8; IQR 21, 3, respectively)^4^. These results show that the FRE score was very variable in the study and that the ChatGPT responses were very difficult to read. In a study of radiology reports, although FRE and FKGL levels were slightly higher (means difficult to read), they were still below the values in our study (38.0 ± 11.8 vs. 40.52 ± 9.81, 10.4 ± 1.9 vs. 12.58 ± 1.66, respectively)^43^. Similar to the literature, the average FRE and FKGL scores found in our study indicate that the responses generated by ChatGPT are very difficult to read and can only be understood by university graduates.

The responses were found to be at the "college freshman" level according to the Gunning fog index and at the "college student" level according to the automated readability index (ARI) (13.63 ± 1.54 and 15.04 ± 1.97, respectively). According to the ARI index, the answers can only be understood by those aged 18–22 and older (maximum level). According to other readability formulas, the Coleman-Liau index and the Dale-Chall index, the responses given by ChatGPT were found to be at the "collage" level (not easy to read, difficult) (14.24 ± 1.90 and 10.03 ± 0.62, respectively). In the SMOG index, which is frequently used in the field of health, the average readability level is 14.61 ± 1.45, indicating that the texts produced by ChatGPT are quite difficult to read. In another study on urology patients, the readability scores of the texts produced by ChatGPT were evaluated, and the mean SMOG index was found to be 8.7 ± 2.1. In the same study (8th or 9th grade), the mean FKE and FKGR scores of the summary texts produced by ChatGPT were also high (means difficult to read) (56.0 ± 13.7 and 10.0 ± 2.4, respectively)^44^.

## Conclusions

This study has shown that the readability levels of the responses generated by ChatGPT are "collage" and above and are difficult to read. Of course, the fact that the subject we tested belongs to a high-level field such as medicine is also effective in reaching this conclusion. However, considering that many people of different age groups and educational levels use ChatGPT to get information about lung cancer, it should be considered that the readability level will be high along with the reliability of the answers given and may be misunderstood or not understood at all. Perhaps in the near future, the ChatGPT can be programmed to produce responses that are appropriate for people of different educational and age groups. It is also clear that there is a need for more extensive and advanced research on a wider range of medical topics.

### Supplementary Information

Supplementary Information.

## Data Availability

The data that support the findings of this study are included in the article/Supplementary Material. Further inquiries can be directed to the corresponding author.
